# The complete mitochondrial genome of *Leptomastidea bifasciata* (Hymenoptera: Chalcidoidea: Encyrtidae) and phylogenetic analysis

**DOI:** 10.1080/23802359.2025.2555459

**Published:** 2025-09-03

**Authors:** Zhi-Peng Chen, Wen-Jian Li, Hong-Xia Hou, Guo-Hao Zu

**Affiliations:** ^a^ College of Horticulture and Landscape, Tianjin Agricultural University, Tianjin, P. R. China; ^b^ Jiangsu Provincial Key Laboratory of Coastal Wetland Bioresources and Environmental Protection, School of Wetland, Yancheng Teachers University, Yancheng, P. R. China; ^c^ College of Chemical Engineering and Biotechnology, Xingtai University, Xingtai, P. R. China

**Keywords:** Encyrtidae, *Leptomastidea bifasciata*, mitochondrial genome, phylogeny, parasitoid wasp

## Abstract

*Leptomastidea* (Hymenoptera, Encyrtidae) are parasitoids of agricultural/forestry mealybug pests. This study first sequences and annotates *L. bifasciata*’s complete mitochondrial genome (15,768 bp), which contains 13 PCGs, 22 tRNAs, 2 rRNAs. All 13 PCGs start with ATN (ATT, ATG, ATA); most end with TAA, except ND1 (TAG). Phylogenetic analysis shows *L. bifasciata* is sister to *Anagyrus* spp.

## Introduction

Encyrtidae is one of the largest families of superfamily Chalcidoidea (Insecta: Hymenoptera), with currently over 4830 species in 518 genera (Zhang et al. 2024). Encyrtidae comprises two subfamilies: Encyrtinae and Tetracneminae. Due to the diminutive body size of its species, DNA extraction is challenging, resulting in most available molecular data being fragmentary and complete mitochondrial genomes remaining limited. Such data are predominantly concentrated in Encyrtinae, with only three species in Tetracneminae having known complete mitochondrial genomes (Ma et al. 2019; Zhang et al. 2024). The species examined herein, *Leptomastidea bifasciata*, belongs to Tetracneminae. Most members of *Leptomastidea* are economically significant, primarily parasitizing Pseudococcidae; additionally, *Leptomastidea acanthococci* Myartseva 1978 and *L. bifasciata* (Mayr 1876) have also been reported to parasitize Eriococcidae (Trjapitzin 2009). Thus far, *Leptomastidea* contains 24 described species, and 7 species have been reported from China (Zu and Li 2017; Japoshvili et al. 2016). The complete mitochondrial genome of *L. bifasciata* was assembled and analyzed, and its coverage depth map is shown in the (supplementary materials Fig. S1). These results help fill the existing data gap and provide valuable molecular resources for future studies on the taxonomy, phylogeny, and evolutionary biology of this species and related groups.

## Materials and methods

**Figure 1.**
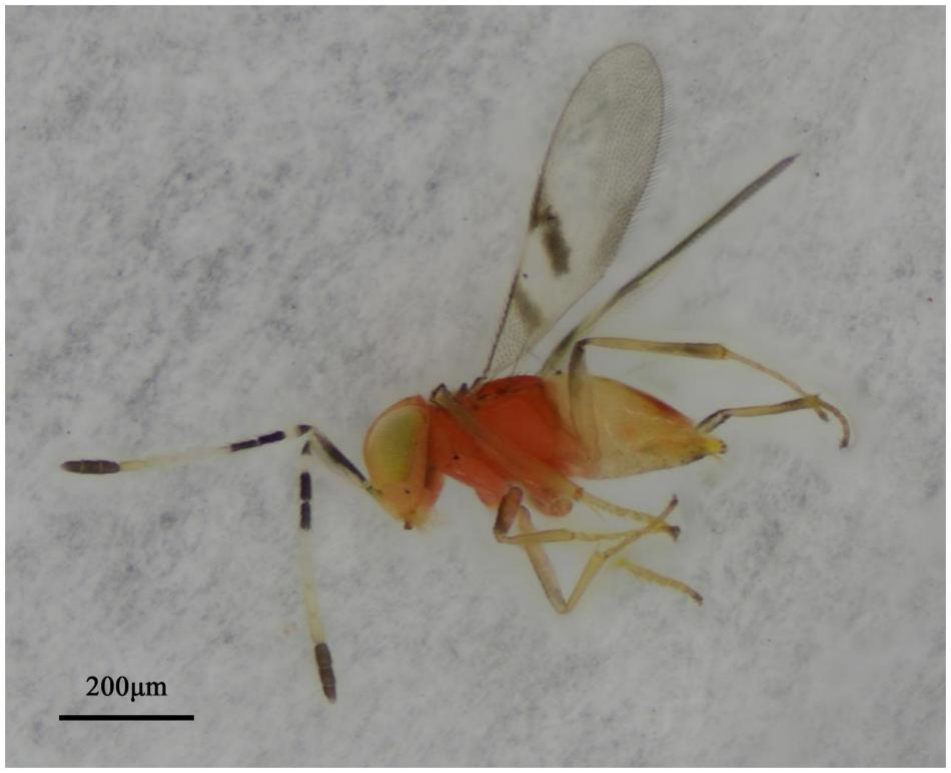
Morphological characteristics of *Leptomastidea bifasciata*. The adult specimen exhibits key diagnostic features: mesosoma orange-red, forewing with 2 transverse fuscous stripes, and antenna with 6 funicle segments (a characteristic of *Leptomastidea*). Scale bar: 200 μm. This photograph was taken by Zhipeng Chen at the Entomology Laboratory of Tianjin Agricultural University.

Specimens of *L. bifasciata* (Figure 1) were collected from Xiqing District, Tianjin, China (39.092 N, 117.102 E) in September 2022. They were stored in absolute ethanol at −40 °C, and vouchered as 220914Lep at the Insect Herbarium of Tianjin Agricultural University (Dr. Guohao Zu, zuguohao@tjau.edu.cn). Total genomic DNA was extracted from one specimen of *L. bifasciata* using the DNeasy Blood & Tissue Kit (Qiagen, Hilden, Germany). Qualified libraries were pooled and sequenced on the Illumina HiSeq 6000 platform (Illumina, San Diego, CA, USA) using the 150 bp paired-end strategy at Novogene Bioinformatics Technology Co., Ltd (Beijing, China), following standard protocols based on effective library concentration and required data volume, generating 6 Gb of raw data.

Following the receipt of raw sequencing data, quality control was executed using fastp v.0.23.4 (Chen et al. 2018) to generate clean reads with a Phred quality score ≥ Q30. Mitochondrial genome assembly was independently performed using two bioinformatics pipelines: MitoZ v.3.6 (Meng et al. 2019) and Get Organelle v.1.7.7.0 (Jin et al. 2020). Secondary structures of transfer RNAs (tRNAs) were predicted using the Galaxy platform (Afgan et al. 2016) and visually validated with VARNA v.3.9 (Darty et al. 2009). The mitogenome map was generated using CGview Server. Nucleotide composition and relative synonymous codon usage (RSCU) of protein-coding genes (PCGs) were analyzed with MEGA v. 11.0.13 (Tamura et al. 2021).

Phylogenetic analyses were performed using 16 mitogenomes from two families of Chalcidoidea, including 15 Encyrtidae species and 1 species from Eulophidae (designated as the outgroup) (Table S1). Phylogenetic trees were constructed using both maximum likelihood (ML) and Bayesian inference (BI) methods. Each protein-coding gene (PCG) was individually aligned *via* the MAFFT v.7 online service with the L-INS-i strategy, followed by optimization using MACSE (Ranwez et al. 2018). The aligned sequences were trimmed with GBlocks and concatenated into a combined PCG dataset using PhyloSuite v.1.2.3 (Talavera and Castresana 2007; Zhang et al. 2020). The optimal nucleotide substitution model was selected based on the Bayesian information criterion (BIC) using ModelFinder v.2.2.0 (Kalyaanamoorthy et al. 2017). For BI analysis, MrBayes v.3.2.7a was employed with four chains and two independent runs of 2,000,000 generations, with sampling conducted every 1,000 generations. The first 25% of trees were discarded as burn-in, and convergence was confirmed when the average standard deviation of split frequencies was <0.01 and the potential scale reduction factor (PSRF) approached 1.0. ML analysis was implemented in IQ-TREE v.2.2.0 (Nguyen et al. 2015) with 1,000 bootstrap replicates under the standard bootstrap approximation.

## Results

**Figure 2.**
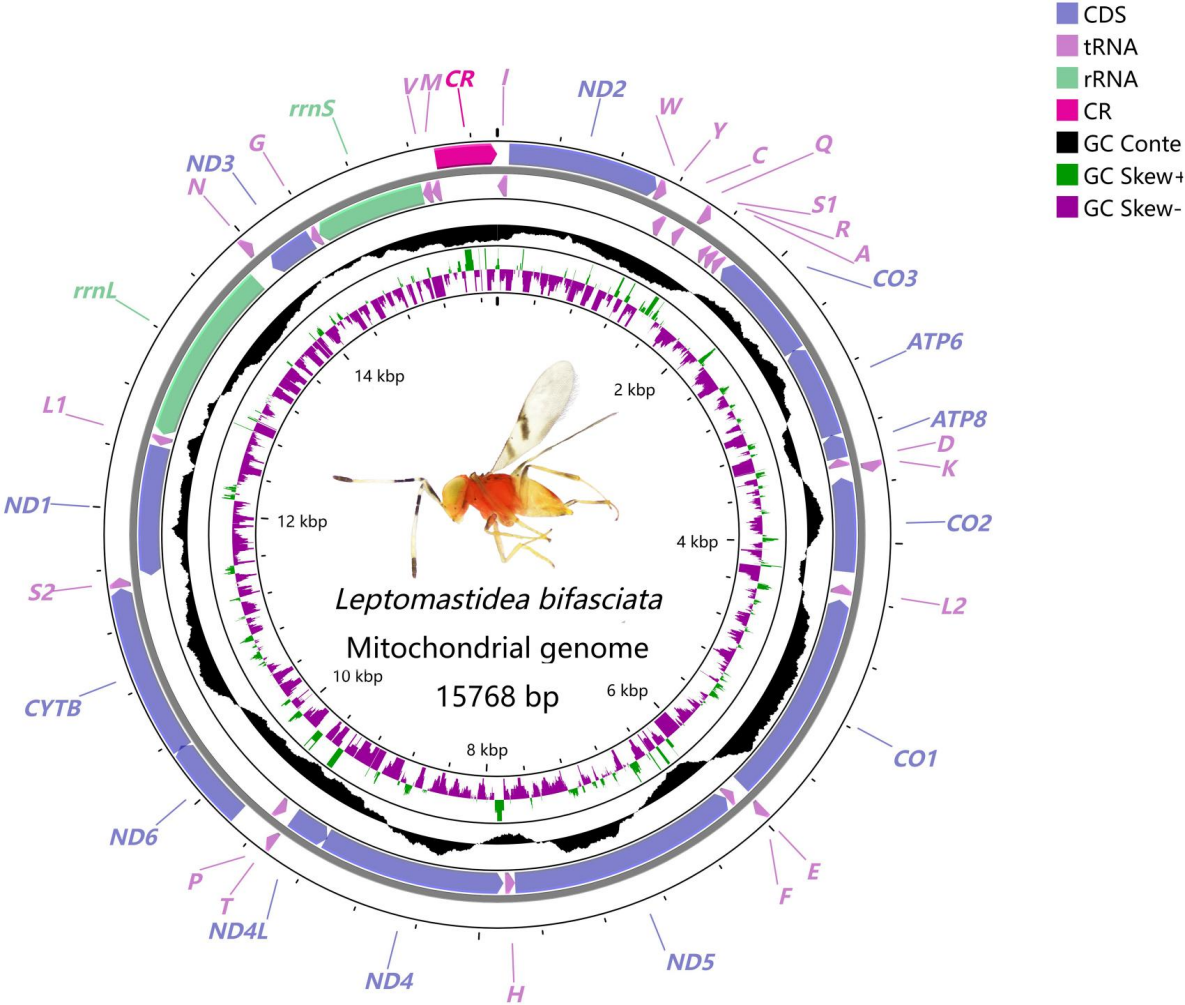
Circular map of the mitochondrial genome of *Leptomastidea bifasciata* (with a total length of 15,768 bp). The outer ring shows the positions of protein-coding genes (PCGs, labeled in blue), transfer RNA genes (tRNAs, labeled with single letters), ribosomal RNA genes (rRNAs: *rrnS* and *rrnL*, labeled in green), and the control region (CR, labeled in red). The inner rings display GC content (gray histogram), GC skew (positive values in purple, negative in orange), and genome length markers (kbp).

The complete mitogenome of *L. bifasciata* has been submitted to GenBank with the accession number OR790123. It is a cyclic molecule of 15,768 bp in length (Figure 2) and consists of 13 PCGs, 22 tRNAs, two rRNAs, and a control region (CR). The mitochondrial gene arrangement of *L. bifasciata* is identical to that of other species in the tribe Anagyrini. The 13 PCGs of this species have a total length of 11,154 bp, 10 of which are encoded on the N-chain (*ND3*, *CO3*, *ATP6*, *ATP8*, *CO2*, *CO1*, *ND5*, *ND4*, *ND4L*, and *ND1*), with the remaining three located on the J-chain (*ND2*, *ND6*, *CYTB*). All PCGs of *L. bifasciata* use ATN as start codons (ATA, ATC, ATG, and ATT). Specifically, *ND1* initiates with ATA; *ATP6*, *CO1*, *CO3*, *CYTB*, *ND4*, and *ND6* start with ATG; and *ATP8*, *CO2*, *ND2*, *ND3*, *ND4L*, and *ND5* use ATT as the start codon. With regard to termination codons, all PCGs terminate with TAG or TAA: *ND1* uses TAG, while the remaining PCGs use TAA (Table S2). The whole mitochondrial genome of *L. bifasciata* has an AT content of 88.0%, exhibiting an AT bias. Among the 13 PCGs, *ATP8* has the highest AT content (92.1%) and *CO1* the lowest (76.4%), with the PCGs overall exhibiting AT biases (Table S3).

Of the 22 tRNA secondary structures, only *trnS1* lacked the DHU arm and failed to form a stable cloverleaf structure; the remaining 21 tRNAs exhibited the typical cloverleaf structure. In these tRNA secondary structures, besides canonical Watson-Crick pairings (A-U, C-G), Non-Canonical pairings (e.g. G-U) were also observed. Specifically, G-U pairings occurred 15 times in total, involving *trnQ*, *trnR*, *trnA*, *trnD*, *trnL2*, *trnF*, *trnH*, *trnP*, *trnG*, and *trnV* (Figure S2).

## Discussion and conclusion

Here, we first assembled and annotated the complete mitogenome of *L. bifasciata* (GenBank accession: OR790123). It is 15,768 bp long with an AT content of 85.0%. This study provides key molecular data for evolutionary and phylogeographic analyses of *L. bifasciata* and a basis for investigating phylogenetic relationships within Encyrtidae.

**Figure 3.**
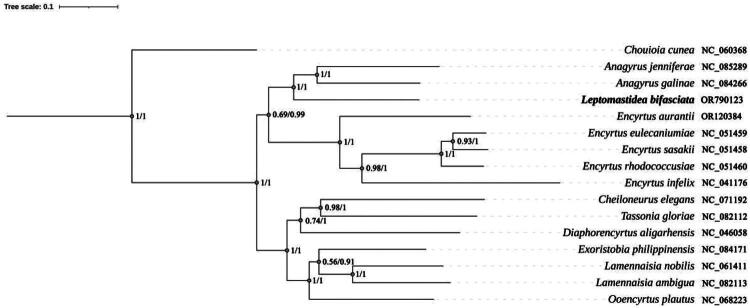
The phylogenetic tree was constructed based on 13 PCGs by Bayesian inference and maximum likelihood methods. The number at each node indicate the posterior probability and bootstrap values resulting from the analyses (ML on the left and BI on the right). The species name and corresponding NCBI accession number of each species involved in this phylogenetic tree are shown on the right. The sequences used are referenced in Table S1.

Phylogenetic analysis (Figure 3) showed that *L. bifasciata* is closely related to *Anagyrus jenniferae* Noyes and Hayat (1994) and *Anagyrus galinae* (Myartseva 1982), and forms a monophyletic group. Pseudococcidae species are common hosts of both *L. bifasciata* and *Anagyrus* species, supporting the phylogenetic results.

Traditionally, *Leptomastidea* and *Anagyrus* are classified in Anagyrini; our phylogenetic results further clarify their relationships. Comparison of its mitogenome with related species clarifies its taxonomic status in Encyrtidae, and provides insights into the evolutionary history of *Leptomastidea* and related groups, aiding future improvements to Encyrtidae phylogeny.

## Supplementary Material

Supplementary Material Table.doc

Supplementary Material Figure.docx

Leptomastidea bifasciata .pdf

## Data Availability

The genome sequence data supporting the results of this study are publicly available at GenBank of NCBI (https://www.ncbi.nlm.nih.gov/) under accession number OR790123. The associated BioProject, SRA, and Bio-Sample numbers are PRJNA1145561, SRR30201849, and SAMN43072041 respectively.
