# Supplementary material for: The complete mitochondrial genome of Leptomastidea bifasciata (Hymenoptera: Chalcidoidea: Encyrtidae) and phylogenetic analysis
Source: Mitochondrial DNA B Resour. 2025 Sep 3;10(10):919–22. doi: 10.1080/23802359.2025.2555459 (PMC12412320; doi:10.1080/23802359.2025.2555459)

Supplementary Table S1. List of all 16 species, GenBank accession numbers and references for sequences used to construct phylogenetic trees (Figure 3).

| **Species** | **Accession number** | **References** |
| --- | --- | --- |
| *Leptomastidea bifasciata* | OR790123 | This study |
| *Chouioia cunea* | NC_060368 | Tang et al. 2021 |
| *Anagyrus jenniferae* | NC_085289 | Direct Submission |
| *Anagyrus galinae* | NC_084266 | Direct Submission |
| *Encyrtus aurantii* | OR120384 | Direct Submission |
| *Encyrtus eulecaniumiae* | NC_051459 | Rudoy et al. 2022 |
| *Encyrtus sasakii* | NC_051458 | Rudoy et al. 2022 |
| *Encyrtus rhodococcusiae* | NC_051460 | Rudoy et al. 2022 |
| *Encyrtus infelix* | NC_041176 | Xiong et al. 2019 |
| *Cheiloneurus elegans* | NC_071192 | Direct Submission |
| *Tassonia gloriae* | NC_082112 | Direct Submission |
| *Diaphorencyrtus aligarhensis* | NC_046058 | Du et al. 2019 |
| *Exoristobia philippinensis* | NC_084171 | Chi et al. 2024 |
| *Lamennaisia nobilis* | NC_061411 | Direct Submission |
| *Lamennaisia ambigua* | NC_082113 | Direct Submission |
| *Ooencyrtus plautus* | NC_068223 | Xing et al. 2022 |

**GenBank References**

Tang X, Lyu B, Lu H, Tang J, Meng R, Cai B. 2021. The mitochondrial genome of a parasitic wasp, *Chouioia cunea* Yang (Hymenoptera: Chalcidoidea: Eulophidae) and phylogenetic analysis. Mitochondrial DNA B Resour. 6(3):81-98.

Rudoy A, Zhu C, Ferrari R, Zhang Y. 2022. Integrative taxonomy based on morphometric and molecular data supports recognition of the three cryptic species within the *Encyrtus sasakii* complex (Hymenoptera, Encyrtidae). J Hymenopt Res. 90:129-152.

Xiong M, Zhou QS, Zhang YZ. 2019. The complete mitochondrial genome of *Encyrtus infelix* (Hymenoptera: Encyrtidae). Mitochondrial DNA B. 4(1):114-115.

Du Y, Song X, Liu X, Zhong B. 2019. Mitochondrial genome of *Diaphorencyrtus aligarhensis* (Hymenoptera: Chalcidoidea: Encyrtidae) and phylogenetic analysis. Mitochondrial DNA B Resour. 4(2):3190-3191.

Chi ZH, Zhang CH, Chen ZP, Cui WY, Wang HY, Zu GH. 2024. The complete mitochondrial genome of *Exoristobia philippinensis* (Hymenoptera: Chalcidoidea: Encyrtidae) and phylogenetic analysis. Mitochondrial DNA Part B: Resources. 9(2):1357-1358.

Xing ZP, Liang X, Wang X, Hu HY, Huang YX. 2022. Novel gene rearrangement pattern in mitochondrial genome of *Ooencyrtus plautus* Huang & Noyes, 1994: new gene order in Encyrtidae (Hymenoptera, Chalcidoidea). Zookeys. 1124:1-21.

Supplementary Table. S2 Gene organization of the mitochondrial genome of *Leptomastidea bifasciata*.


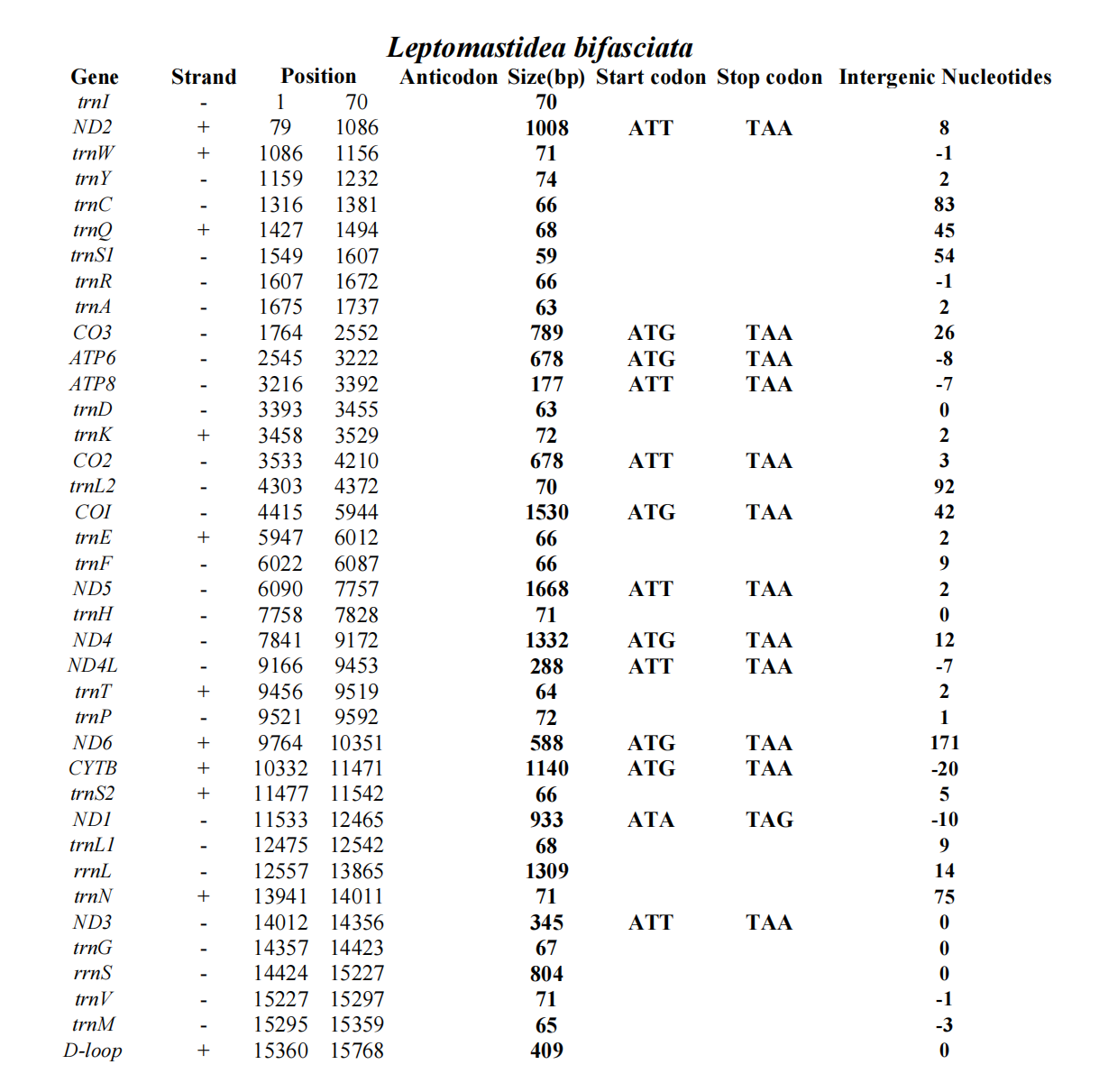


Supplementary Table. S3 Nucleotidae features of the mitochondrial genome of *Leptomastidea bifasciata*.


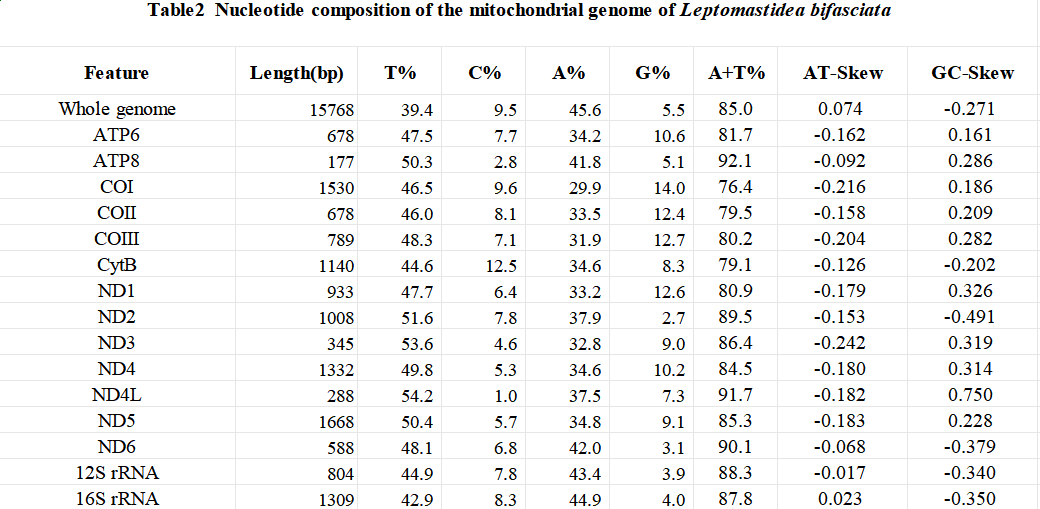

Supplement: Supplementary Material Table.doc [file TMDN_A_2555459_SM8820.doc]
