# Supplementary material for: The complete mitochondrial genome of Leptomastidea bifasciata (Hymenoptera: Chalcidoidea: Encyrtidae) and phylogenetic analysis
Source: Mitochondrial DNA B Resour. 2025 Sep 3;10(10):919–22. doi: 10.1080/23802359.2025.2555459 (PMC12412320; doi:10.1080/23802359.2025.2555459)
Supplement: Supplementary Material Figure.docx [file TMDN_A_2555459_SM8819.docx]

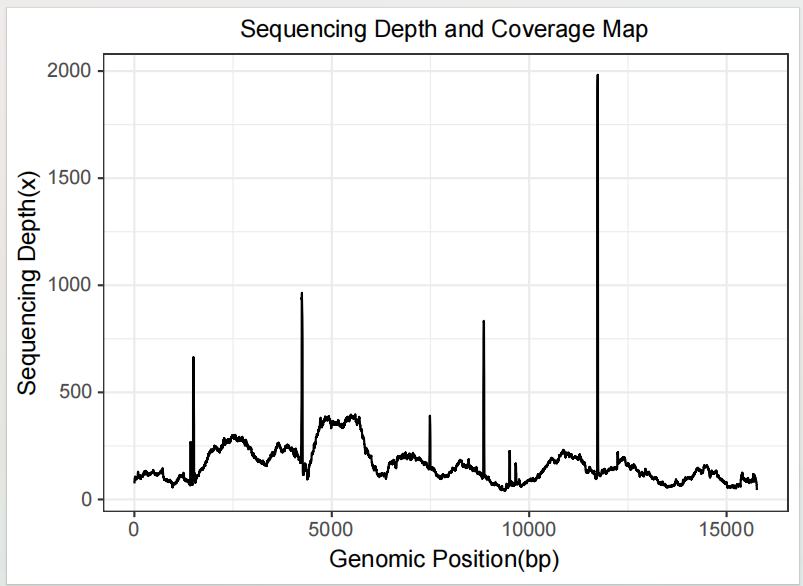


Supplementary Fig. S1 Sequencing Depth and Coverage Map of *Leptomastidea bifasciata*.


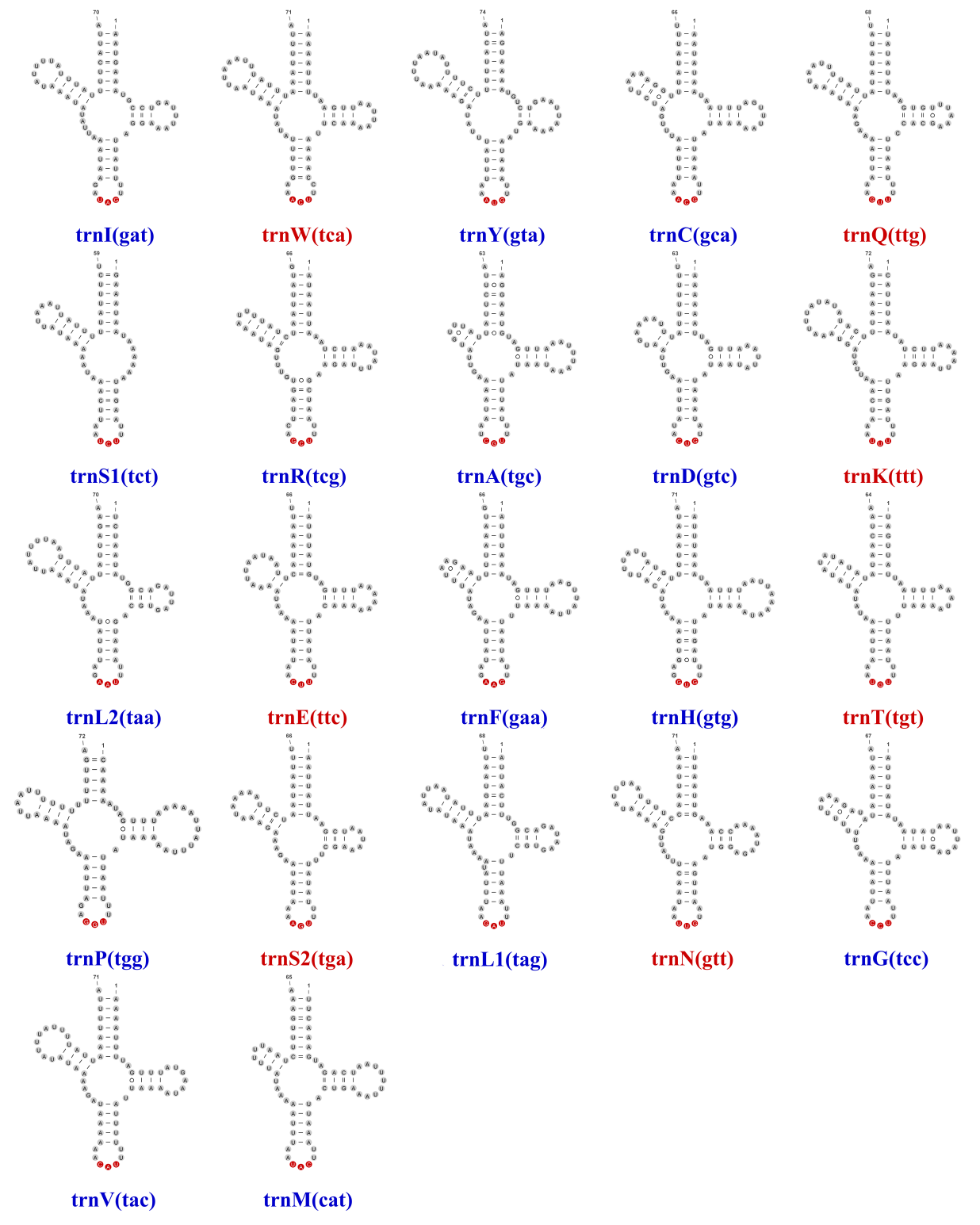


Supplementary Fig. S2 The tRNA secondary structures of *Leptomastidea bifasciata*.
