# Supplementary material for: The complete mitochondrial genome of Leptomastidea bifasciata (Hymenoptera: Chalcidoidea: Encyrtidae) and phylogenetic analysis
Source: Mitochondrial DNA B Resour. 2025 Sep 3;10(10):919–22. doi: 10.1080/23802359.2025.2555459 (PMC12412320; doi:10.1080/23802359.2025.2555459)
Supplement: Leptomastidea bifasciata .pdf [file TMDN_A_2555459_SM8818.pdf]

The complete mitochondrial genome of *Leptomastidea bifasciata* (Hymenoptera: Chalcidoidea: Encyrtidae) and phylogenetic analysis

Zhi-Peng Chen[a], Wen-Jian Li [b], Hong-Xia Hou[c, d], Guo-Hao Zu[a, d]

[a]College of Horticulture and Landscape, Tianjin Agricultural University, Tianjin, 300392, P. R. China

[b]Jiangsu Provincial Key Laboratory of Coastal Wetland Bioresources and Environmental Protection, School of Wetland, Yancheng Teachers University, Yancheng, 224007, P. R. China.

[c]College of Chemical Engineering and Biotechnology, Xingtai University, Xingtai, Hebei, 054000, P. R. China

[d]Corresponding author: Hong-Xia Hou (houhongxia003@126.com), Guo-Hao Zu (zuguohao@tjau.edu.cn)

ABSTRACT

Leptomastidea, a genus of Encyrtidae (Hymenoptera, Chalcidoidea), represents a successful group of parasitoid insects that attack various mealybug pests of agricultural and forestry plants. This study presents the complete mitochondrial genome of *L. bifasciata* for the first time, which was sequenced and annotated. The genome is 15,768 bp in length, encoded 13 protein-coding genes (PCGs), 22 transfer RNA genes (tRNAs), and 2 ribosomal RNA genes (rRNAs). All 13 PCGs were initiated by the ATN (ATT, ATG, and ATA) codon, terminated with the stop codon TAA except for ND1 which ends with TAG. Phylogenetic analysis showed that *L. bifasciata* has a sister relationship with *Anagyrus* spp..

KEYWORDS

Encyrtidae; *Leptomastidea bifasciata*; mitochondrial genome; phylogeny

Introduction

Encyrtidae is one of the largest families of superfamily Chalcidoidea (Insecta: Hymenoptera), with currently over 4830 species in 518 genera (Zhang et al. 2024). Encyrtidae comprises two subfamilies: Encyrtinae and Tetracneminae. Due to the diminutive body size of its species, DNA extraction is challenging, resulting in most available molecular data being fragmentary and complete mitochondrial genomes remaining limited. Such data are predominantly concentrated in Encyrtinae, with only 3 species in Tetracneminae having known complete mitochondrial genomes (Ma et al. 2019, Zhang et al. 2024). The species examined herein, *Leptomastidea bifasciata*, belongs to Tetracneminae. Most members of *Leptomastidea* are economically significant, primarily parasitizing Pseudococcidae; additionally, *Leptomastidea acanthococci* Myartseva 1978 and *L. bifasciata* (Mayr 1876) have also been reported to parasitize Eriococcidae (Trjapitzin 2009). Thus far, *Leptomastidea* contains 24

described species, and 7 species have been reported from China (Zu and Li 2017, Japoshvili et al. 2016). The complete mitochondrial genome of *L. bifasciata* was assembled and analyzed, and its coverage depth map is shown in the supplementary materials (Fig. S1). These results help fill the existing data gap and provide valuable molecular resources for future studies on the taxonomy, phylogeny, and evolutionary biology of this species and related groups.

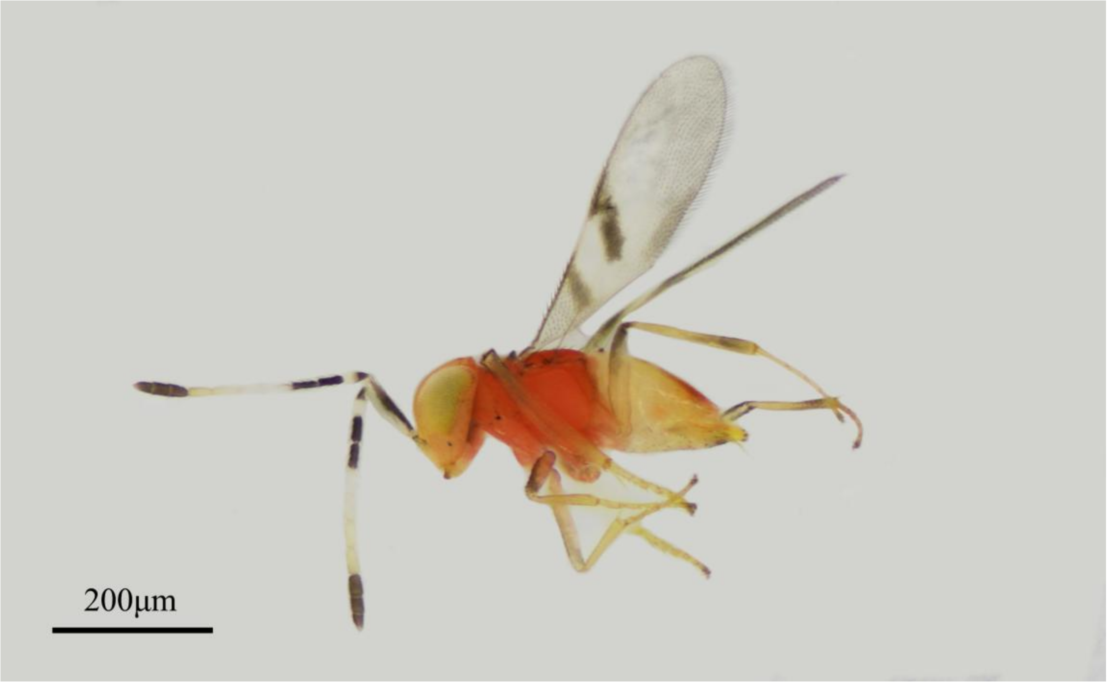

Fig. 1 Morphological characteristics of *Leptomastidea bifasciata*. The adult specimen exhibits key diagnostic features: mesosoma orange-red, forewing with 2 transverse fuscous stripes, and antenna with 6 funicle segments (a characteristic of *Leptomastidea*). Scale bar: 200 μm. This photograph was taken by Zhipeng Chen at the Entomology Laboratory of Tianjin Agricultural University.

Materials and Methods

Specimens of *L. bifasciata* (Fig. 1) were collected from Xiqing District, Tianjin, China (39.092 N, 117.102 E) in September 2022. They were stored in absolute ethanol at -40°C, and vouchered as 220914Lep at the Insect Herbarium of Tianjin Agricultural University (Dr. Guohao Zu, zuguohao@tjau.edu.cn). Total genomic DNA was extracted from one specimen of *L. bifasciata* using the DNeasy Blood & Tissue Kit (Qiagen, Hilden, Germany). Qualified libraries were pooled and sequenced on the Illumina HiSeq 6000 platform (Illumina, San Diego, CA, USA) using the 150 bp paired-end strategy at Novogene Bioinformatics Technology Co., Ltd (Beijing, China), following standard protocols based on effective library concentration and required data volume, generating 6 Gb of raw data.

Following the receipt of raw sequencing data, quality control was executed using fastp v.0.23.4 (Chen et al. 2018) to generate clean reads with a Phred quality score ≥ Q30. Mitochondrial genome assembly was independently performed using two bioinformatics pipelines: MitoZ v.3.6 (Meng et al. 2019) and Get Organelle v.1.7.7.0 (Jin et al. 2020). Secondary structures of transfer RNAs (tRNAs) were predicted using

the Galaxy platform (Afgan et al. 2016) and visually validated with VARNA v.3.9 (Darty et al. 2009). The mitogenome map was generated using CGview Server. Nucleotide composition and relative synonymous codon usage (RSCU) of protein-coding genes (PCGs) were analyzed with MEGA v. 11.0.13 (Tamura et al. 2021).

Phylogenetic analyses were performed using 16 mitogenomes from two families of Chalcidoidea, including 15 Encyrtidae species and 1 species from Eulophidae (designated as the outgroup) (Table S1). Phylogenetic trees were constructed using both maximum likelihood (ML) and Bayesian inference (BI) methods. Each protein-coding gene (PCG) was individually aligned via the MAFFT v.7 online service with the L-INS-i strategy, followed by optimization using MACSE (Ranwez et al. 2018). The aligned sequences were trimmed with GBlocks and concatenated into a combined PCG dataset using PhyloSuite v.1.2.3 (Talavera and Castresana 2007, Zhang et al. 2020). The optimal nucleotide substitution model was selected based on the Bayesian information criterion (BIC) using ModelFinder v.2.2.0 (Kalyaanamoorthy et al. 2017). For BI analysis, MrBayes v.3.2.7a was employed with four chains and two independent runs of 2,000,000 generations, with sampling conducted every 1,000 generations. The first 25% of trees were discarded as burn-in, and convergence was confirmed when the average standard deviation of split frequencies was <0.01 and the potential scale reduction factor (PSRF) approached 1.0. ML analysis was implemented in IQ-TREE v.2.2.0 (Nguyen et al. 2015) with 1,000 bootstrap replicates under the standard bootstrap approximation.

Results

The complete mitogenome of *L. bifasciata* has been submitted to GenBank with the accession number OR790123. It is a cyclic molecule of 15,768 bp in length (Fig. 2) and consists of 13 PCGs, 22 tRNAs, two rRNAs, and a control region (CR). The mitochondrial gene arrangement of *L. bifasciata* is identical to that of other species in the tribe Anagyrini. The 13 PCGs of this species have a total length of 11,154 bp, 10 of which are encoded on the N-chain (*ND3*, *CO3*, *ATP6*, *ATP8*, *CO2*, *CO1*, *ND5*, *ND4*, *ND4L*, and *ND1*), with the remaining three located on the J-chain (*ND2*, *ND6*, *CYTB*). All PCGs of *L. bifasciata* use ATN as start codons (ATA, ATC, ATG, ATT). Specifically, *ND1* initiates with ATA; *ATP6*, *CO1*, *CO3*, *CYTB*, *ND4*, and *ND6* start with ATG; and *ATP8*, *CO2*, *ND2*, *ND3*, *ND4L*, and *ND5* use ATT as the start codon. With regard to termination codons, all PCGs terminate with TAG or TAA: *ND1* uses TAG, while the remaining PCGs use TAA (Table S2). The whole mitochondrial genome of *L. bifasciata* has an AT content of 88.0%, exhibiting an AT bias. Among the 13 PCGs, *ATP8* has the highest AT content (92.1%) and *CO1* the lowest (76.4%), with the PCGs overall exhibiting AT biases (Table S3).

Of the 22 tRNA secondary structures, only *trnS1* lacked the DHU arm and failed to form a stable cloverleaf structure; the remaining 21 tRNAs exhibited the typical cloverleaf structure. In these tRNA secondary structures, besides canonical Watson-Crick pairings (A-U, C-G), Non-Canonical pairings (e.g., G-U) were also observed. Specifically, G-U pairings occurred 15 times in total, involving *trnQ*, *trnR*,

trnA, *trnD*, *trnL2*, *trnF*, *trnH*, *trnP*, *trnG*, and *trnV* (Fig. S2).

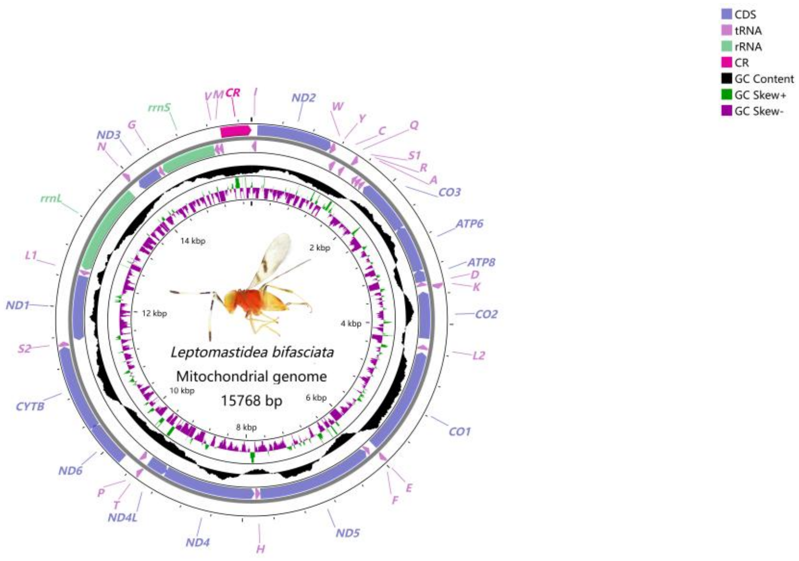

Fig. 2 Circular map of the mitochondrial genome of *Leptomastidea bifasciata* (with a total length of 15,768 bp). The outer ring shows the positions of protein-coding genes (PCGs, labeled in blue), transfer RNA genes (tRNAs, labeled with single letters), ribosomal RNA genes (rRNAs: *rrnS* and *rrnL*, labeled in green), and the control region (CR, labeled in red). The inner rings display GC content (gray histogram), GC skew (positive values in purple, negative in orange), and genome length markers (kbp).

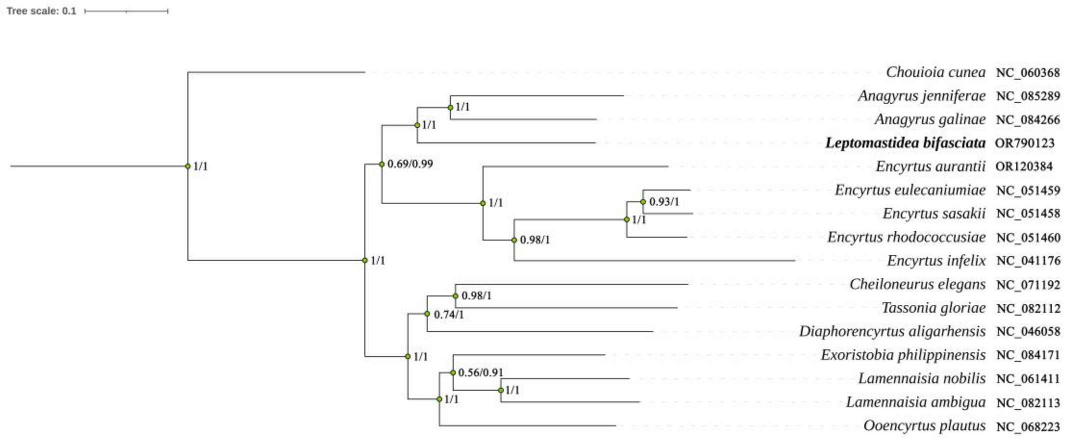

Fig. 3 The phylogenetic tree was constructed based on 13 PCGs by Bayesian inference and maximum likelihood methods. The number at each node indicate the posterior probability and bootstrap values resulting from the analyses (ML on the left and BI on the right). The species name and corresponding NCBI accession number of each species involved in this phylogenetic tree are shown on the right. The sequences used are referenced in Table S1.

Discussion and conclusion

Here, we first assembled and annotated the complete mitogenome of *L. bifasciata* (GenBank accession: OR790123). It is 15,768 bp long with an AT content

of 85.0%. This study provides key molecular data for evolutionary and phylogeographic analyses of *L. bifasciata* and a basis for investigating phylogenetic relationships within Encyrtidae.

Phylogenetic analysis (Fig. 3) showed that *L. bifasciata* is closely related to *Anagyrus jenniferae* Noyes & Hayat 1994 and *Anagyrus galinae* (Myartseva 1982), and forms a monophyletic group. Pseudococcidae species are common hosts of both *L. bifasciata* and *Anagyrus* species, supporting the phylogenetic results.

Traditionally, *Leptomastidea* and *Anagyrus* are classified in Anagyrini; our phylogenetic results further clarify their relationships. Comparison of its mitogenome with related species clarifies its taxonomic status in Encyrtidae, and provides insights into the evolutionary history of *Leptomastidea* and related groups, aiding future improvements to Encyrtidae phylogeny.

Acknowledgements
We thank Tao Wang for his assistance with software application and analysis.
We thank Lu-Jie An for her valuable assistance in processing the insect photographs for this study using Photoshop.

Author contributions

Zhipeng Chen and Guohao Zu made significant contributions to data analysis and manuscript preparation. Guohao Zu and Wenjian Li were responsible for species selection, while Zhipeng Chen undertook species photography, experimental implementation, and data analysis. Wenjian Li, Guohao Zu, and Hongxia Hou critically revised the intellectual content of the manuscript. Guohao Zu and Hongxia Hou participated in the conception and design of the study, and gave final approval for the version to be published. All authors agree to be accountable for all aspects of the work.

Ethics statement

Leptomastidea bifasciata used in this study complies with international ethical requirements. Our practices are in full compliance with Chinese legislation, as well as adhering to pertinent guidelines, policies, and regulations. Notably, the collection site is not designated as a nature reserve, and the acquisition of specimens was executed without causing harm to the local ecology. Furthermore, all experiments were carried out under the vigilant supervision and with the explicit permission of Tianjin Agricultural University.

Disclosure statement

No potential conflict of interest was reported by the authors. The authors are responsible for the content and writing of the paper.

Funding
This research was funded by the National Natural Science Foundation of China (Grant number: 32200375), the Natural Science Foundation of Hebei Province, China (C2024108006), and Tianjin Basic Research Fund for Scientific Research in Higher Education Institutions (2021KJ115).

ORCID
Hongxia Hou https://orcid.org/0000-0002-0203-8809

Guohao Zu https://orcid.org/0000-0002-9892-2171
Zhipeng Chen https://orcid.org/0009-0006-9947-5074
Wenjian Li https://orcid.org/0009-0004-9790-3608

Data availability statement

The genome sequence data supporting the results of this study are publicly available at GenBank of NCBI (https://www.ncbi.nlm.nih.gov/) under accession number OR790123. The associated BioProject, SRA, and Bio-Sample numbers are PRJNA1145561, SRR30201849 , and SAMN43072041 respectively.

References

Zhang CH, Wang HY, Wang Y, Chi ZH, Liu YS, Zu GH. 2024. The first two complete mitochondrial genomes for the genus *Anagyrus* (Hymenoptera, Encyrtidae) and their phylogenetic implications. ZooKeys. 1206:81–98. doi:10.3897/zookeys.1206.121923.

Ma X, Zhang Y, Li X, Li S. 2019. The mitochondrial genome of *Aenasius arizonensis* (Hymenoptera: Encyrtidae) with novel gene order. Mitochondrial DNA Part B. 4:2023–2024. doi:10.1080/23802359.2019.1617052.

Myartseva, MM. 1978. New species of encyrtids (Hymenoptera, Encyrtidae) from Turkmenistan. Entomol Obozr. 57(2):377–381.

Mayr G. 1876. Die europäischen Encyrtiden. Verhandlungen der zoologisch-botanischen Gesellschaft in Wien. 25:675–778.

Trjapitzin VA. 2009. Review of species of the genus *Leptomastidea* Mercet, 1916 (Hymenoptera, Encyrtidae) of the world, with description of a new species from Montenegro and with separation of a new genus from Turkmenia. Entomol Obozr. 88(1):164–176. doi:10.1134/s0013873809020122.

Zu GH, Li CD. 2017. Descriptions of *Leptomastidea angulipennis* sp. nov. and two known species of *Leptomastidea* (Hymenoptera: Encyrtidae), with a key to Chinese species. ZooKeys. 28(1):189–194. doi:10.1007/s11676-016-0309-z.

Japoshvili G, Higashiura Y, Kamitani S. 2016. A review of Japanese Encyrtidae (Hymenoptera), with descriptions of new species, new records and comments on the types described by Japanese authors. Acta Entomol Mus Nat Pragae. 56(1):345–401. doi:zoobank.org/urn:lsid:zoobank.org:pub:D7B9D474-65C3-4358-8057-DD672465241B.

Chen SF, Zhou YQ, Chen YR, Gu J. 2018. fastp: An ultra-fast all-in-one FASTQ preprocessor. Bioinformatics. 34(17): i884–i890. doi:10.1093/bioinformatics/bty560.

Meng GL, Li YY, Yang CT, Liu SL. 2019. MitoZ: A toolkit for animal mitochondrial genome assembly, annotation and visualization. Nucleic Acids Res. 47(11): e63. doi:10.1093/nar/gkz173.

Jin JJ, Yu WB, Yang JB, Song Y, dePamphilis CW, Yi TS, Li DZ. 2020. GetOrganelle: A fast and versatile toolkit for accurate de novo assembly of organelle genomes. Genome Biol. 21(1):241. doi:10.1186/s13059-020-02154-5.

Afgan E, Baker D, Batut B, van den Beek M, Bouvier D, Cech M, Chilton J, Clements D, Coraor N, Grüning B, Guerler A, Hillman-Jackson J, Hiltemann S,

Jalili V, Rasche E, Soranzo N, Turaga N, Taylor J, Nekrutenko A, Goecks J. 2016. The Galaxy platform for accessible, reproducible and collaborative biomedical analyses: 2016 update. Nucleic Acids Res. 44(W1): W3–W10. doi:10.1093/nar/gkw343.

Darty K, Denise A, Ponty Y. 2009. VARNA: Interactive visualization of RNA secondary structure. Bioinformatics. 25(15):1974–1975. doi:10.1093/bioinformatics/btp250.

Tamura K, Stecher G, Kumar S. 2021. MEGA11: Molecular Evolutionary Genetics Analysis version 11. MOL BIOL EVOL. 38(7):3022–3027. doi:10.1093/molbev/msab120.

Ranwez V, Harispe S, Dufayard JF, Koncz C, Champagnat F, Ponty Y, Lartillot N, Galtier N, Thompson JD. 2018. MACSE v2: Toolkit for the alignment of coding sequences accounting for frameshifts and stop codons. MOL BIOL EVOL. 35(9):2210–2215. doi:10.1093/molbev/msy159.

Talavera G, Castresana J. 2007. Improvement of phylogenies after removing divergent and ambiguously aligned blocks from protein sequence alignments. Syst Biol. 56(4):564–577. doi:10.1080/10635150701472164.

Zhang D, Gao FL, Jakovlić I, Zou H, Zhang J, Li WX, Wang GT. 2020. PhyloSuite: An integrated and scalable desktop platform for streamlined molecular sequence data management and evolutionary phylogenetics studies. Mol Ecol Resour. 20(1):348–355. doi:10.1111/1755-0998.13096.

Kalyaanamoorthy S, Minh BQ, Wong TKF, von Haeseler A, Jermiin LS. 2017. ModelFinder: Fast model selection for accurate phylogenetic estimates. Nat Methods. 14(6):587–589. doi:10.1038/nmeth.4285.

Nguyen LT, Schmidt HA, von Haeseler A, Minh BQ. 2015. IQ-TREE: A fast and effective stochastic algorithm for estimating maximum-likelihood phylogenies. MOL BIOL EVOL. 32(1):268–274. doi:10.1093/molbev/msu300.

Noyes JS, Hayat M (1994) Oriental mealybug parasitoids of the Anagyrini (Hymenoptera: Encyrtidae). CAB International, Wallingford. 554 pp. doi:10.1017/s0007485300033071.

Myartseva SN. 1982. New species of the parasitic Hymenoptera, Encyrtidae from the Amu Darya Tugai. Izv Akad Nauk Turkm SSR (Ser Biol Nauk). 2:40–48.
